# Maternal-fetal outcomes of pregnant women with type 1 diabetes treated with continuous subcutaneous insulin infusion or multiple daily injections during pregnancy – A Brazilian health care referral center cohort study

**DOI:** 10.20945/2359-4292-2022-0483

**Published:** 2023-06-19

**Authors:** Juliana Ogassavara, Patricia Medici Dualib, Rosiane Mattar, Sérgio Atala Dib, Bianca de Almeida-Pititto

**Affiliations:** 1 Universidade Federal de São Paulo Programa de Pós-graduação em Endocrinologia e Metabologia São Paulo SP Brasil Programa de Pós-graduação em Endocrinologia e Metabologia, Universidade Federal de São Paulo, São Paulo, SP, Brasil; 2 Universidade Federal de São Paulo Departamento de Medicina São Paulo SP Brasil Departamento de Medicina, Universidade Federal de São Paulo, São Paulo, SP, Brasil; 3 Universidade Federal de São Paulo Departamento de Obstetrícia São Paulo SP Brasil Departamento de Obstetrícia, Universidade Federal de São Paulo, São Paulo, SP, Brasil; 4 Universidade Federal de São Paulo Departamento de Medicina Preventiva São Paulo SP Brasil Departamento de Medicina Preventiva, Universidade Federal de São Paulo, São Paulo, SP, Brasil

**Keywords:** Continuous subcutaneous insulin infusion, maternal-fetal outcomes, multiple daily injections, type 1 diabetes mellitus, pregnancy in diabetics

## Abstract

**Objective:**

Pregnant women with type 1 diabetes (T1D) have an increased risk of maternal-fetal complications. Regarding treatment, continuous subcutaneous insulin infusion (CSII) has advantages compared to multiple daily injections (MDI), but data about the best option during pregnancy are limited. This study's aim was to compare maternal-fetal outcomes among T1D patients treated with CSII or MDI during pregnancy.

**Subjects and methods:**

This study evaluated 174 pregnancies of T1D patients. Variables of interest were compared between the groups (CSII versus MDI), and logistic regression analysis was performed (p < 0.05).

**Results:**

Of the 174 included pregnancies, CSII was used in 21.3% (37) and MDI were used in 78.7% (137). HbA1c values improved throughout gestation in both groups, with no difference in the first and third trimesters. The frequency of cesarean section was significantly higher in the CSII group [94.1 *vs.* 75.4%, p = 0.017], but there was no significant difference in the frequency of other complications, such as miscarriage, premature delivery and preeclampsia. The mean birth weight and occurrence of neonatal complications were also similar, except for the proportion of congenital malformations, which was significantly lower in the CSII group [2.9 *vs.* 15.6%, p = 0.048]. In regression analysis, the association of CSII with cesarean section and malformations lost significance after adjusting for HbA1c and other covariates of interest.

**Conclusion:**

In this study, we observed a higher frequency of cesarean section and a lower occurrence of congenital malformations in the CSII group, but the adjusted results might indicate that these associations are influenced by glycemic control.

## INTRODUCTION

Diabetes mellitus (DM) represents an important and growing public health problem worldwide. In 2021, the International Diabetes Federation estimated that 537 million people (10.5% of the world population) were living with DM. Type 1 DM (T1D) corresponds to 5-10% of all DM cases, and the number of children and adolescents living with T1D increases annually, with Brazil having the third highest number of patients in the world (1).

As T1D is usually diagnosed at an earlier age, many women of reproductive age are affected. It is known that pregnant women with preexisting DM are at increased risk of maternal-fetal complications, including spontaneous abortions, fetal malformations, macrosomia, preeclampsia, prematurity, cesarean section, and the worsening of complications related to DM itself, in addition to long-term repercussions on their offspring, such as a higher incidence of DM, obesity and cognitive alterations (2-15). Intensive glycemic control before conception and during pregnancy is essential to reduce the occurrence of these adverse outcomes (16-21).

To achieve glycemic control goals, the current treatment of T1D relies on insulin therapy through multiple doses of insulin (MDI) or a continuous insulin infusion system (CSII). However, data on the best option for the treatment of pregnant women with T1D are conflicting; to date, there are no Brazilian studies on maternal-fetal repercussions in this specific population, and data from other developing countries are sparse (22-27). Thus, the main objective of this study was to compare the occurrence of maternal-fetal outcomes in pregnant women with T1D who were treated with CSII or MDI at a referral center of the public health network in Sao Paulo, Brazil.

## SUBJECTS AND METHODS

### Study population and design

This was a cohort study enrolling women previously diagnosed with T1D who were followed up at the Diabetes Center of Universidade Federal de Sao Paulo, Brazil, during their prenatal care from January 2008 to December 2021. The medical information of these women was collected by the researchers during prenatal care visits and compiled into a database. All women who met the eligibility criteria were invited to participate (n = 178 patients, corresponding to 213 pregnancies).

**Figure 1:**
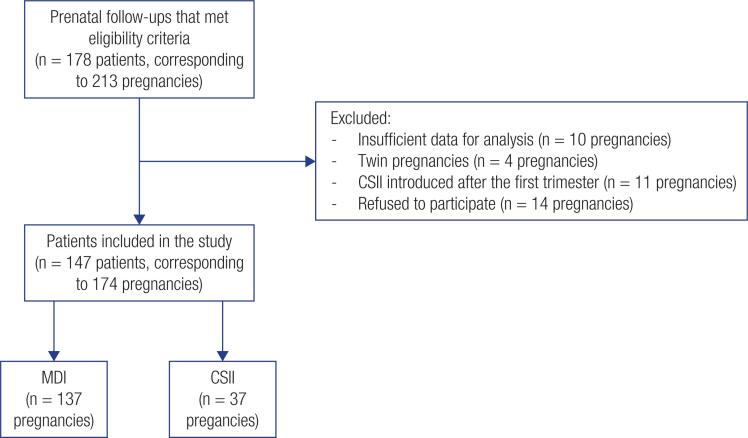
Flowchart of patient selection.

The eligibility criteria were as follows: pregnant women with a diagnosis of T1D; pregnant women aged over 18 years at the time of prenatal care; and pregnant women receiving insulin therapy through MDI or CSII introduced before pregnancy or at the latest, in the first trimester of pregnancy. The prescription/indication for treatment with MDI or CSII was made by the routine follow-up physician without intervention by the researchers of this study. Patients with insufficient data for analysis, those with twin pregnancies or those for whom CSII was introduced after the first trimester were excluded from the study (n= 39 pregnancies). After exclusion, a total of 147 women were included in this study, totaling 174 different pregnancies analyzed (Figure 1).

The institutional ethics committee of the Universidade Federal de Sao Paulo approved the study, and all participants signed a consent form.

### Data collection and study variables of interest

Weight was obtained on a digital scale (Rice Lake, São Paulo) with an accuracy of 100 g, and height was determined with an accuracy of 0.5 cm. Pregestational weight was self-reported, and pregestational body mass index (BMI) was calculated (kg/m^2^). Blood pressure measurements were obtained using a mercury sphygmomanometer.

Blood samples were collected in routine care to perform laboratory tests. Plasma glucose was determined by the glucose oxidase method, glycated hemoglobin (HbA1c) levels were determined by high-performance liquid chromatography and thyroid stimulating hormone (TSH) levels were determined by chemiluminescence. The concentrations of total cholesterol, high-density lipoprotein cholesterol (HDL-c) and triglycerides were determined by enzymatic colorimetric methods, and samples were processed in an automatic analyzer. Low-density lipoprotein cholesterol (LDL-c) and very low-density lipoprotein cholesterol (VLDL-c) concentrations were obtained by difference using the Friedewald equation.

Personal pathological and obstetrical history, comorbidities associated with DM, other chronic diseases and chronic complications of T1D were assessed during medical prenatal care visits, as well as maternal/fetal complications, delivery type, gestational age at delivery and birth weight. Maternal complications were defined as hypertension, preeclampsia, and hypothyroidism, and fetal complications were defined as malformations, admission to the neonatal intensive care unit (NICU), hypoglycemia, hyperbilirubinemia, respiratory distress and neonatal death.

### Statistical analysis

The sample was divided according to the type of treatment into the CSII group and the MDI group (exposure variables). Maternal-fetal outcomes (HbA1c levels, delivery type, gestational age at delivery, birth weight and maternal or fetal complications) were evaluated according to the type of treatment. Continuous variables are presented as the mean (standard deviation) when parametric or as the median (interquartile range) when nonparametric, and categorical variables are presented as the frequency (percentage). Clinical and laboratory variables were compared according to the treatment received. Student's t test was used for parametric continuous variables, the Mann-Whitney test was used for nonparametric continuous variables, and Pearson's chi-squared or the Fischer test was used for categorical variables. Logistic regression analyses were performed considering the outcomes that were significantly different between the exposure (CSII) and comparison (MDI) groups as dependent variables (cesarean section and congenital malformations) and treatment with CSII as the independent variable, adjusted for the covariates of interest (HbA1c levels during pregnancy, age, duration of DM, pregestational BMI, preeclampsia, gestational hypertension and hypothyroidism). Statistical Package for the Social Sciences®, v 22.0 (SPSS Incorporation, 2000) was used, and p < 5% was considered statistically significant.

## RESULTS

A total of 174 pregnancies were evaluated, and the patients had a mean age of 26.7 (5.4) years. The average duration of diabetes was 14.3 (6.9) years, and the mean pregestational BMI was 24.4 (3.6) kg/m². Regarding the type of treatment, 137 (78.7%) participants were treated with MDI, while 37 (21.3%) were treated with CSII.

Among these two groups, statistically significant differences were not shown in mean age, baseline BMI, weight gain during pregnancy, levels of lipid profile variables or the frequency of chronic hypertension, but hypothyroidism was more prevalent in the CSII group than the MDI group [32.4 *vs*. 17.5%, p = 0.047]. The CSII group also had a longer mean (SD) duration of T1D [18.0 (6.5) *vs*. 13.3 (6.7) years, p < 0.001], but no differences were shown in the prevalence of chronic complications (retinopathy and nephropathy) (Table 1).

**Table 1:** Maternal characteristics according to mode of insulin administration

	MDI (n = 137)	CSII (n = 37)	p value
Age (years)	26.4 (5.3)	27.8 (5.7)	0.163
Diabetes duration (years)	13.3 (6.7)	18.0 (6.5)	0.0002
Pre-pregnancy BMI (kg/m^2^)	24.4 (3.6)	24.5 (3.7)	0.796
Weight gain during pregnancy (kg)	12.7 (4.9)	13.1 (5.2)	0.686
Multiparity, n (%)	52 (38.0)	19 (51.4)	0.141
Diabetic nephropathy, n (%)	24 (27.3)	7 (28)	0.943
Diabetic retinopathy, n (%)π	25 (24.5)	5 (16.1)	0.462
Chronic hypertension, n (%)π	12 (8.8)	5 (13.5)	0.364
Hypothyroidism, n (%)	24 (17.5)	12 (32.4)	0.047
Smoking patients, n (%)π	3 (2.2)	1 (2.7)	1.000
First trimester's HbA1c (%)	8.8 (1.8)	8.3 (1.7)	0.122
Second trimester's HbA1c (%)	7.4 (1.3)	7.1 (1.2)	0.378
Third trimester's HbA1c (%)	7.1 (1.0)	6.9 (0.8)	0.611
Total cholesterol (mg/dL)	184.8 (43.0)	178.3 (58.0)	0.471
HDL cholesterol (mg/dL)	65.0 (17.3)	63.9 (18.0)	0.755
LDL cholesterol (mg/dL)	98.7 (32.2)	95.3 (43.7)	0.616
Triglycerides (mg/dL)#	83 (63-134)	68 (51-105)	0.091
TSH (mUI/L)	1.95 (1.32)	1.87 (0.96)	0.740

Results are given as mean (SD), median (IQR) # or n (%).

Student's t test used for parametric continuous variables, Mann-Whitney test for nonparametric continuous variables

# and Pearson's Chi-squared or Fischer test

π for categorical variables.

Abbreviations: BMI, body mass index; MDI, multiple daily injection; CSII, continuous subcutaneous insulin infusion.

HbA1c levels improved throughout gestation in both groups, but there was no difference in the mean (SD) HbA1c values in the first [8.3 (1.7) *vs*. 8.8 (1.8) %, p = 0.122], second [7.1 (1.2) *vs*. 7.4 (1.3) %, p = 0.378], or third trimesters of pregnancy [6.9 (0.8) *vs*. 7.1 (1.0) %, p = 0.611] when comparing the CSII and MDI groups, respectively (Table 1).

The frequency of cesarean section was significantly higher in the CSII group [94.1 *vs*. 75.4%, p = 0.017], but there was no significant difference in the frequency of other complications, such as miscarriage, premature delivery and preeclampsia. There was also no difference in gestational age at birth or the proportion of full-term and preterm births, but both groups had a greater predisposition to prematurity, with a mean gestational age at delivery of 34.9 (4.8) weeks in the CSII group and 35.8 (3.4) weeks in the MDI group (p = 0.611) (Table 2).

**Table 2:** Maternal and neonatal outcomes according to mode of insulin administration

	MDI	CSII	p value
Gestational age at delivery (weeks)	35.8 (3.4)	34.9 (4.8)	0.611
Births at full term, n (%)	56 (45.5)	13 (37.1)	0.573
Late preterm births (34-37 weeks), n (%)	51 (41.5)	18 (51.4)	
Preterm births (≤33 + 6 weeks), n (%)	16 (13)	4 (11.4)	
Miscarriages (<22 weeks), n (%)π	6 (4.4)	2 (5.4)	0.678
Cesarean section, n (%)	92 (75.4)	32 (94.1)	0.017
Preeclampsia, n (%)	11 (8.4)	6 (16.7)	0.146
Gestational hypertension, n (%)π	3 (2.3)	3 (8.3)	0.115
Birth weight (g)	2988 (728)	2999 (848)	0.941
Small for gestational age, n (%)	8 (6.7)	5 (14.7)	0.175
Large for gestational age, n (%)	23 (19.3)	9 (26.5)	
Composite adverse neonatal outcome, n (%)	103 (84.4)	31 (88.6)	0.541
Perinatal death, n (%)π	7 (5.7)	1 (2.9)	0.685
Hyperbilirubinemia, n (%)	53 (43.4)	19 (54.3)	0.256
Respiratory distress, n (%)	34 (27.9)	13 (37.1)	0.291
Hypoglycemia, n (%)	48 (39.3)	10 (28.6)	0.244
Intensive care unit admission, n (%)	39 (32.0)	12 (34.3)	0.796
Congenital malformation, n (%)π	19 (15.6)	1 (2.9)	0.048

Results are given as mean (SD) or n (%).

Student's t test used for continuous variables and Pearson's Chi-squared or Fischer test

π for categorical variables.

Abbreviations: MDI, multiple daily injection; CSII, continuous subcutaneous insulin infusion.

There was no significant difference in birth weight or in the rates of small for gestational age (SGA) and large for gestational age (LGA) births between the two groups, or in the frequencies of neonatal complications, such as perinatal death, hyperbilirubinemia, respiratory distress, hypoglycemia and NICU admission, except for the proportion of congenital malformations, which was significantly lower in the CSII group [2.9 *vs*. 15.6%, p = 0.048] (Table 2). We observed that 20 babies presented a total of 21 malformations, as follows: congenital heart diseases (n = 13), neural tube defects (n = 3), caudal regression syndrome (n = 2), kidney malformation (n = 1) and congenital limb defects (n = 2).

**Figure 2:**
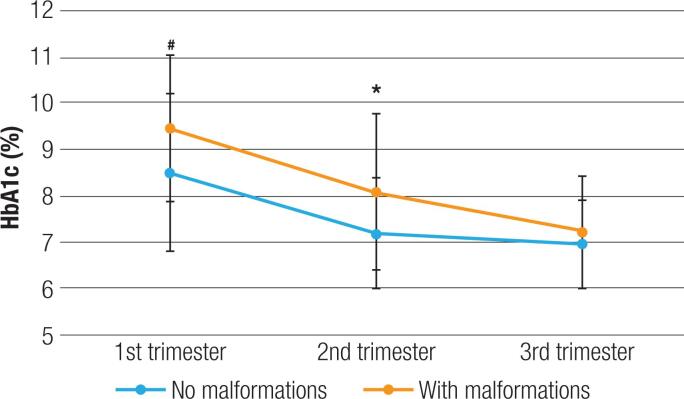
Mean HbA1c per trimester in patients with and without congenital malformations.

When comparing the HbA1c levels of the mothers who had babies with congenital malformations, there was a trend toward higher HbA1c levels in these patients in the first trimester [9.5 (1.6) *vs*. 8.5 (1.7) %, p = 0.078] and the second trimester [8.1 (1.7) *vs*. 7.2 (1.2) %, p = 0.018], the latter with statistical significance (Figure 2).

Student's t test comparing mean HbA1c in patients with and without congenital malformations in each trimester. #For the comparison in first trimester, p = 0.08. *For the comparison in the second trimester, p < 0.05.

In logistic regression analyses, treatment with CSII was significantly and borderline associated with cesarean section and malformations in crude analyses (Model 1), respectively. However, CSII lost its association with both outcomes after adjusting for HbA1c in the first adjusted model (Model 2) and became nonsignificant after adjusting for other covariates of interest (Model 3), as shown in Table 3.

**Table 3:** Association of use of CSII with cesarean section and congenital malformations

	Cesarean section		Congenital malformations	
	OR (95% CI)	p value	OR (95% CI)	p value
Model 1	5.22 (1.18-23.08)	0.029	0.16 (0.02-1.24)	0.079
Model 2	3.52 (0.77-16.13)	0.106	0.29 (0.04-2.51)	0.266
Model 3	2.67 (0.52-13.67)	0.239	0.35 (0.04-3.25)	0.355

Analyzed with logistic regression.

Model 1 – Crude OR.

Model 2 – Cesarean section: adjusted for third trimester's HbA1c/Congenital malformations: adjusted for second trimester's HbA1c.

Model 3 – Cesarean section: model 2 plus age, DM duration, pre-pregnancy BMI, preeclampsia and gestational hypertension/Congenital malformations: model 2 plus age, DM duration, pre-pregnancy BMI and hypothyroidism.

## DISCUSSION

The rate of maternal-fetal complications in our cohort was similar between the CSII and MDI groups, except for cesarean section, which was more frequent in the CSII group, and the rate of congenital malformations, which was higher in the MDI group.

Despite CSII has a number of advantages compared to MDI, such as greater flexibility of use, a more precise titration of the basal insulin dose and the type and size of the prandial boluses, a greater reduction of hypoglycemic episodes, improved glycemic control and improved quality of life, the results of this study are in agreement with the findings of other authors, whose studies evaluating pregnant women with T1D showed conflicting results or did not demonstrate major differences in maternal–fetal outcomes between the two treatment modalities (22-27).

For example, the most recent meta-analysis showed a lower HbA1c level with CSII *vs*. MDI in the first trimester (WMD: -0.45%; 95% CI: -0.62, -0.27), but this difference decreased in subsequent trimesters. Compared to MDI, CSII resulted in higher gestational weight gain (WMD: 1.02 kg; 95% CI: 0.41, 1.62) and lower daily insulin dose requirements in the first (SMD: -0.46; 95% CI: -0.68, -0.24) and subsequent trimesters. Moreover, infants from mothers in the CSII group were more likely to be LGA (RR: 1.16; 95% CI: 1.07, 1.24) and less likely to be SGA (RR: 0.66; 95% CI: 0.45; 0.97) [23].

Another recent study involving 209 pregnant women, with 95 being treated with MDI and 114 treated with CSII, demonstrated no differences in the daily dose of insulin (both total and per kg of body weight), body mass index or weight gain. The 1st and 2nd trimester HbA1c levels were lower among the CSII group [6.83 (1.38) *vs*. 7.52 (2.11) %, p = 0.01 and 6.17 (0.9) *vs*. 6.57 (1.12) %, p = 0.009, respectively], while the 3rd trimester HbA1c level as well as the total change in HbA1c were comparable. There were also no differences in the gestational age at delivery, the mode of delivery, neonatal birth weight, or the rates of macrosomia, LGA or SGA. A higher Apgar score was noted among infants born to women in the CSII group [8.63 (1.63) *vs*. 8.03 (2.49), p = 0.047); however, the proportion of neonates with an Apgar score lower than 7 points was similar (27).

In the present study, the characteristics of the groups were similar, except for the duration of DM, which was significantly longer in patients using insulin pumps. This difference must be considered, as the duration of DM is associated with an increased risk of complications related to DM. The CSII group had a significantly higher cesarean section rate than the MDI group, but the rate in both groups was quite remarkable (94.1% and 75.4%, respectively), which is partly explained by the fact that Brazil has the second highest cesarean section rate in the world (55.7%) (28). In addition, a longer duration of DM has been associated with a higher risk of obstetric complications (4,29,30), which could contribute to the higher cesarean section rate seen in this group. However, even with a longer duration of DM, we observed that the group that was treated with CSII had similar rates of retinopathy, nephropathy, preeclampsia, miscarriage, premature delivery and most neonatal complications. Thus, we hypothesize that treatment with CSII might have a potential protective effect in this sample of patients with a longer duration of diabetes.

In addition, it should be noted that in Brazil, access to treatment with CSII is very limited due to socioeconomic conditions. Most patients in the public health system can only receive CSII via a high-cost drug process or judicial process, and even private health insurance plans do not cover this type of treatment. Thus, those who have CSII treatment approved and funded by the government generally have more severe DM, with glycemic variability and hypoglycemia that are difficult to control even after optimized treatment with insulin analogs (31,32). Considering this context, the fact that the CSII group had outcomes similar to those to the MDI group can be interpreted as positive in our opinion.

In our cohort, it is important to note that patients were not in adequate glycemic control in early pregnancy in either group. Unfortunately, we do not have information on whether the pregnancies were planned or not, but recently, our group published a study that evaluated the intention of pregnancy and its influence on the HbA1c profile before and during pregnancy in women with previously diagnosed DM, which included some participants from the current study. The results showed that glycemic control did not differ between the groups that did or did not intend to become pregnant, with a mean prepregnancy HbA1c value of 9.3%, even with 83.3% reporting having received guidance on the importance of glucose control and contraception before becoming pregnant (33).

Despite suboptimal glycemic control in our sample, there was an improvement throughout pregnancy in both groups, showing that adequate follow-up plays an essential role in this issue, regardless of the route of insulin administration. It is important to emphasize that these patients were followed-up in a specialized center of public health in Brazil, with a multidisciplinary team engaged in optimizing the management of diabetes and frequent visits at the center during gestation.

Regarding congenital malformations, a significant difference in the prevalence of this outcome was found between the CSII and MDI groups. It is known that the presence of pregestational DM significantly increases the risk of congenital malformations (34) and that the worse the glycemic control, the greater the risk (35). An interesting finding of our study is that, despite similar HbA1c values, the CSII group had lower rates of congenital malformations than the MDI group, which could indicate that factors other than HbA1c might be protective, such as possible lower glycemic variability in the CSII group. Corroborating this hypothesis, the subanalysis that compared the groups with and without malformations showed that patients with this outcome had higher HbA1c values, with statistical significance in the second trimester. There was probably no significant difference in the first trimester due to the low number of patients with HbA1c values during this period of pregnancy.

In logistic regression analyses, treatment with CSII was significantly and borderline associated with cesarean section and malformations in crude analyses, but the association was lost for both outcomes after the first adjustment for HbA1c values during pregnancy and persisted without significant association when adjusting for other confounding variables; this demonstrated that other characteristics, such as glucose control (HbA1c values during pregnancy), age, the duration of DM, pregestational BMI, preeclampsia, gestational hypertension and hypothyroidism, could play an important role in these outcomes.

Limitations of the study include the observational design and the low number of patients treated with CSII compared with those treated with MDI, which affects the power of the study. In addition, it was not possible to evaluate the total daily dose of insulin used, preconception HbA1c values and other parameters of glycemic control, such as time in range and the presence of hypoglycemia during pregnancy, since a small portion of patients had access to CGM in this sample. Furthermore, the evaluation was based on data from 2008 to 2021, and therefore, many patients used insulin pumps without sensor integration that are not comparable to the new models that are currently used.

The strength of this study is that it represents a real-life cohort with fewer recall and information biases, and is the first Brazilian study on this topic. Most of the published studies were carried out in Europe or in the United States, which does not represent the profile of our population and the public health system in Brazil.

In conclusion, in this study, we observed a higher frequency of cesarean section and a lower occurrence of congenital malformations in the CSII group, which lost statistical significance after adjusting for possible confounders. The higher cesarean section rate might represent more severe cases of DM, since these patients had a longer duration of DM and the profile of insulin pump users in Brazil is usually a more challenging DM control. Although there was no difference in HbA1c values when compared to the MDI group, the lower frequencies of malformations in the CSII group might be mediated by glucose control during early pregnancy, as we observed a trend toward worse HbA1c trajectory values during pregnancy in women whose infants had malformations than in those whose infants did not have malformations. The evaluation of other parameters of glycemic control, such as glycemic variability and time in range, might clarify this hypothesis in future studies regarding the comparison of CSII and MDI treatments for T1D in pregnancy. There was no difference in other maternal-fetal outcomes between the two groups.
